# Distal adding-on after surgery in Lenke 5C adolescent idiopathic scoliosis: clinical and radiological outcomes

**DOI:** 10.1186/s12891-022-05559-4

**Published:** 2022-06-22

**Authors:** Wenbin Hua, Zhiwei Liao, Wencan Ke, Shuai Li, Xiaobo Feng, Bingjin Wang, Kun Wang, Xinghuo Wu, Yukun Zhang, Yong Gao, Li Ling, Cao Yang

**Affiliations:** 1 Department of Orthopaedics, Union Hospital, Tongji Medical College, Huazhong University of Science and Technology, Wuhan, 430022 China; 2 Department of VIP Clinic, Union Hospital, Tongji Medical College, Huazhong University of Science and Technology, Wuhan, 430022 China

**Keywords:** Adolescent idiopathic scoliosis, Lenke 5C, Surgery, Adding-on, Lower instrumented vertebra, Last barely touched vertebra, Last substantial touched vertebra

## Abstract

**Background:**

To evaluate the incidence and risk factors of postoperative distal adding-on in patients with Lenke 5C adolescent idiopathic scoliosis (AIS). More accurate selection criteria for the lower instrumented vertebra (LIV) should be confirmed to prevent distal adding-on.

**Methods:**

Forty-six patients with Lenke 5C AIS who underwent posterior fusion were enrolled in the study. Patients were allocated into *adding-on* and *no adding-on* groups. Demographic data, clinical data, and radiographic parameters were recorded and compared.

**Results:**

Postoperative distal adding-on occurred in eight patients (17.4%) during follow-up. Demographic data, clinical data, and baseline radiographic parameters of the two groups were not significantly different. The postoperative thoracolumbar (TL) or lumbar (L) Cobb angle, LIV translation, and LIV + 1 translation were higher in the adding-on group than those in the no adding-on group, while the postoperative coronal imbalance of the adding-on group was lower than that of the no adding-on group. The level difference of last barely touched vertebra (LBTV) and last substantial touched vertebra (LSTV) with LIV were higher in the adding-on group than in the no adding-on group.

**Conclusion:**

Postoperative TL/L curve, postoperative LIV translation, postoperative LIV + 1 translation, and postoperative coronal imbalance were determined as risk factors for postoperative distal adding-on in patients with Lenke 5C AIS. Moreover, LIV selection of LBTV-1 or LSTV-1 may cause a higher risk of postoperative distal adding-on.

## Background

Lenke 5C adolescent idiopathic scoliosis (AIS) is defined as a curve with structural thoracolumbar (TL) or lumbar (L) scoliosis [1–3]. Surgery is considered in patients with Lenke 5C AIS with progressive Cobb angles greater than 40°, especially in cases of coronal imbalance or cosmetic demand [1–4]. Posterior fusion with pedicle screws is commonly used to treat Lenke 5C AIS with excellent outcomes [1–3, 5, 6]. Although the Lenke classification is reliable to determine the surgical plan, the determination of the ideal fusion level can be challenging [3–5]. The selection of the lower instrumented vertebra (LIV) in patients with Lenke 5C AIS has been extensively discussed in previous studies, while the criteria remain uncertain [7, 8]. LIV selection is important to achieve optimal correction and to preserve lumbar mobility [8].

It is critical to fuse the less mobile segments to achieve optimal global balance during the treatment for patients with Lenke 5C AIS [3, 9]. The choice of a too proximal LIV may result in distal curve decompensation, while a too distal LIV may cause a needless sacrifice of lumbar motion [10]. According to the “Cobb to Cobb method”, the Lenke 5C curve is usually fused between the upper end vertebra and the lowest end vertebra (LEV) [6, 11]. L3 is commonly selected as the LIV, while longer fusions with an LIV below L3 will result in a greater loss of lumbar function [4].

The postoperative distal adding-on in AIS is usually found with a progressive correction loss due to an increase in either lumbar vertebral deviation or LIV disc angulation [8, 12]. Postoperative distal adding-on in Lenke 5C AIS has aroused increasing attention and remains to be fully investigated [13]. The occurrence rate of postoperative distal adding-on in patients with Lenke 5C AIS is between 2 and 51% [13]. Progressive degeneration of the lumbar spine and aggravated coronal imbalance may increase the probability of revision surgery [13]. The LIV should be located in a stable zone to minimize the risk of postoperative distal adding-on [4, 7, 13, 14].

The purpose of the present study was to evaluate the incidence and risk factors of postoperative distal adding-on in patients with Lenke 5C AIS. Moreover, the LIV selection criteria for these patients were determined and evaluated.

## Methods

### Patients

Between October 2012 and September 2019, all patients with Lenke 5C AIS who underwent posterior fusion and pedicle screw instrumentation were consecutively included. This retrospective case series was approved by the institutional review board of our hospital.

The inclusion criteria were as following: (1) diagnosis of Lenke 5C AIS according to the Lenke classification system; (2) the Cobb angle of the TL/L curve greater than 40°; (3) patient underwent posterior TL fusion surgery with pedicle screw instrumentation; (4) age between 11 and 18 years; and (5) a minimum 24-month follow-up. The exclusion criteria were as following: (1) AIS of other types according to the Lenke classification system, degenerative scoliosis, and other types of spine deformity; (2) loss to follow-up; and (3) previous thoracolumbar surgery or revision surgery.

### LIV Selection Criteria

The LIV selection criteria were as following: (1) the last barely touched vertebra (LBTV) or last substantial touched vertebra (LSTV) touched by the central sacrum vertical line (CSVL); (2) the vertebra crossed by CSVL between two pedicles on the concave bending radiograph; and (3) the vertebra not at the apex of kyphosis. All criteria must have been met, and the LIV was determined. The LBTV or LSTV touched by the CSVL was confirmed on standing radiographs [1, 8, 15].

### Surgical Procedure

All surgical procedures were performed by the same senior surgeon (C.Y.) using the standard posterior fusion technique in the prone position. Via a midline incision, subperiosteal dissection was performed to adequately exposure the posterior elements of the spine. Bilateral pedicle screws were inserted using the free hand technique. Arthrectomy was usually performed at each fusion level. Rod rotation, compression, distraction, and segmental derotation techniques were commonly used to correct the scoliosis [13]. The in situ bending maneuvers were performed where necessary. The facet joints were thoroughly decorticated, then autograft and allograft (Aorui, China) were used for fusion.

Intraoperative multimodal neurophysiological monitoring, including motor evoked potentials and somatosensory evoked potentials, was routinely performed.

### Radiographic Parameters

Radiographic parameters were measured by two independent doctors. Full length anteroposterior (AP) and lateral radiographs of the spine were reviewed prior to surgery, after surgery, and at the final follow-up. Preoperative curve flexibility was evaluated by bending radiographs. The Cobb angles of the TL/L and thoracic (T) curves were measured. The LIV tilt was measured as the angle between the inferior endplate of the LIV and the horizontal line [12]. The LIV disc angle was assessed as the disc angle immediately adjacent to the LIV. LIV translation and LIV + 1 translation were the horizontal offset from the center of the LIV and LIV + 1 to the CSVL, respectively [12]. The coronal imbalance was determined as the horizontal offset of the C7 plumb line from the CSVL [13, 16]. Moreover, stable vertebra, neutral vertebra, lowest end vertebra (LEV), LSTV, and LBTV were identified [17–19].

Postoperative distal adding-on in patients with Lenke 5C AIS was defined as the progressive distal deformity after surgery, with an increase in LIV disc angle of more than 5° or an increase in LIV + 1 translation for more than 5 mm [13, 15].

### Clinical Outcomes

Patients were allocated to an *adding-on group* or *no adding-on group* according to the occurrence of distal adding-on. Demographic data, including age, sex, Risser grade at surgery, and length of follow-up were recorded. Clinical data, including fused levels, operative time, estimated blood loss, and complications, were also recorded. Demographic, clinical, and radiological data of both groups were compared. The SRS-22 questionnaire was administered preoperatively and at the 24-month postoperative follow-up to determine clinical outcomes.

### Statistical Analysis

Data are presented as mean ± standard deviation. SPSS 22.0 (IBM Corp., Armonk, NY, USA) was used to perform the statistical analyses. Normal distribution of the data was assessed using the Kolmogorov–Smirnov test. Nonparametric data were analyzed by the Mann–Whitney U test or the Wilcoxon signed-rank test. A logistic regression analysis was also performed. Statistical significance was set at *P* < 0.05.

## Results

### Patients

A total of 46 patients with Lenke 5C AIS were recruited for this study. Demographic data and baseline radiographic parameters of the included patients are summarized in Table 1. Postoperative distal adding-on occurred in eight patients (17.4%) during follow-up.

**Table 1:** Summary of demographic data and baseline radiographic parameters of the included patients

	**Number**	**Range**
Age at surgery (yrs)	15.4 ± 1.8	12–18
Male: Female	13: 33	-
Risser grade at surgery	4.0 ± 1.1	0–5
Fusion levels	6.1 ± 0.7	5–7
Operative time (min)	204.9 ± 37.2	130–300
Estimated blood loss (ml)	528.5 ± 175.7	300–1000
Follow-up(months)	33.5 ± 16.1	24–72
LIV location		
L2	2	-
L3	24	-
L4	20	-
Baseline radiographic parameters		
TL/L curve(°)	47.7 ± 6.9	40.0–64.0
T curve(°)	25.2 ± 5.9	4.0–46.0
LIV tilt(°)	25.9 ± 7.3	14.0–40.0
LIV disc angle(°)	8.0 ± 4.7	1.0–23.0
LIV translation(mm)	22.4 ± 8.5	7.7–50.0
LIV + 1 translation(mm)	8.5 ± 6.3	0–31.0
Coronal imbalance(mm)	23.0 ± 12.4	0–50.0

*LIV* Lower instrumented vertebra; *TL* Thoracolumbar; *L* Lumbar; *T* Thoracic

There were no significant differences in terms of sex, Risser grade at surgery, operative time, estimated blood loss, fused segments, and follow-up period between the two groups (Table 2).

**Table 2:** Comparison of demographic data and clinical data of the two groups

	**Adding-on group(*n* = 8)**	**No adding-on group(*n* = 38)**	***P* value**
Age at surgery (yrs)	14.1 ± 1.2	15.7 ± 1.8	0.023
Male: Female	2/6	11/27	0.876
Risser grade at surgery	3.8 ± 1.0	4.0 ± 1.1	0.416
Fusion levels	6.1 ± 0.6	6.0 ± 0.7	0.831
Operative time (min)	190.0 ± 30.2	208.0 ± 38.1	0.246
Estimated blood loss (ml)	425.0 ± 138.9	550.3 ± 176.3	0.057
Follow-up(months)	31.5 ± 14.2	25.4 ± 4.7	0.467

^*^Statistically significant, *P* < 0.05

### Radiological Outcomes

The radiographic parameters of both the adding-on group and the no adding-on group are summarized in Table 3. Baseline data of the two groups showed no significant differences.

**Table 3:** Comparison of the radiographic parameters of the two groups

	**Adding-on group(*n* = 8)**	**No adding-on group(*n* = 38)**	***P* value**
TL/L curve(°)			
Preoperative	48.2 ± 8.5	47.6 ± 6.6	0.876
Postoperative	11.1 ± 5.5	6.4 ± 4.3	0.023*
Last follow-up	14.4 ± 8.9	8.1 ± 5.5	0.146
T curve(°)			
Preoperative	30.8 ± 5.6	25.6 ± 8.3	0.065
Postoperative	14.0 ± 6.9	9.8 ± 5.6	0.053
Last follow-up	18.0 ± 10.1	11.0 ± 5.8	0.057
LIV tilt(°)			
Preoperative	26.4 ± 6.2	25.0 ± 5.8	0.639
Postoperative	5.6 ± 3.0	5.0 ± 3.7	0.450
Last follow-up	6.3 ± 3.2	5.3 ± 3.7	0.246
LIV disc angle(°)			
Preoperative	6.6 ± 7.4	8.3 ± 4.0	0.102
Postoperative	3.4 ± 2.4	2.4 ± 1.4	0.384
Last follow-up	7.3 ± 4.2	2.8 ± 1.5	0.007*
LIV translation(mm)			
Preoperative	29.1 ± 11.9	21.0 ± 7.0	0.053
Postoperative	12.1 ± 5.1	8.2 ± 5.3	0.036*
Last follow-up	13.1 ± 6.9	7.2 ± 3.2	0.019*
LIV + 1 translation(mm)			
Preoperative	11.9 ± 9.4	7.8 ± 5.4	0.234
Postoperative	7.3 ± 4.0	3.3 ± 3.5	0.009*
Last follow-up	9.0 ± 5.5	2.8 ± 2.6	0.001*
Coronal imbalance(mm)			
Preoperative	19.4 ± 14.7	23.8 ± 11.9	0.258
Postoperative	10.0 ± 11.2	19.7 ± 13.8	0.042*
Last follow-up	10.6 ± 9.7	12.5 ± 8.0	0.433

^*^Statistically significant, *P* < 0.05; *TL* Thoracolumbar; *L* Lumbar; *T* Thoracic; *LIV* Lower instrumented vertebra

Postoperative TL/L curve, postoperative LIV translation, and postoperative LIV + 1 translation of the adding-on group were significantly higher compared to those in the no adding-on group. The coronal imbalance of the adding-on group was significantly lower than that of the no adding-on group. Postoperative T curve, postoperative LIV tilt, and postoperative LIV disc angle showed no significant differences between the two groups.

At the final follow-up, LIV disc angle, LIV translation, and LIV + 1 translation of the adding-on group were significantly higher than those of the no adding-on group. TL/L curve, T curve, LIV tilt, and coronal imbalance showed no significant differences between the two groups.

The LIV selections of the two groups were compared and are summarized in Table 4. The level difference of LBTV and LSTV with LIV was compared, with LBTV-LIV and LSTV-LIV being significantly higher in the adding-on group compared to the no adding-on group. As a result, when the LIV is selected to be LBTV-1 or LSTV-1, the risk of postoperative distal adding-on was higher.

**Table 4:** Comparison of lower instrumented vertebra selection of the two groups

	**Adding-on group(*n* = 8)**	**No adding-on group(*n* = 38)**	***P* value**
LIV			
L2	0	2	0.154
L3	7	17	
L4	1	19	
LBTV-LIV	0.9 ± 0.4	0.2 ± 0.5	0.005*
LSTV-LIV	1.4 ± 0.5	0.6 ± 0.5	0.008*
NV-LIV	1.4 ± 0.7	1.0 ± 0.7	0.234
SV-LIV	1.9 ± 0.6	1.4 ± 0.6	0.109

^*^Statistically significant, *P* < 0.05; LIV, lower instrumented vertebra

Logistic regression analysis was also performed to evaluate the risk factors of postoperative distal adding-on, and the results are summarized in Table 5.

**Table 5:** Logistic regression analysis of risk factors for postoperative distal adding-on after surgery

Risk factors	B	SE	Wald	*P*
Postoperative TL/L curve	-0.100	0.103	0.934	0.334
Postoperative LIV translation	0.043	0.190	0.052	0.819
Postoperative LIV + 1 translation	-0.331	0.267	1.539	0.215
Postoperative coronal imbalance	0.086	0.050	2.911	0.088

*LIV* Lower instrumented vertebra

### Clinical Outcomes

All domains of the SRS-22 scale showed general improvement (Table 6). The SRS-22 questionnaire scores were not significantly different between the adding-on and no adding-on groups preoperatively and at the 24-month postoperative follow-up (Table 7).

**Table 6:** Comparison of SRS-22 outcome between preoperative and postoperative 24-month follow-up

	**Preoperative**	**Postoperative 24-month follow-up**	**Improvement rate (%)**	**p value**
Function, activity	4.3 ± 0.3	4.4 ± 0.3	2.3	0.021*
Pain	4.0 ± 0.3	4.1 ± 0.3	2.5	0.001*
Self-image, Appearance	3.6 ± 0.4	4.2 ± 0.2	16.7	< 0.001*
Mental Health	4.1 ± 0.2	4.3 ± 0.2	4.9	0.002*
Satisfaction	3.8 ± 0.5	4.3 ± 0.2	13.2	< 0.001*
Total	4.0 ± 0.1	4.2 ± 0.1	5.0	< 0.001*

^*^Statistically significant, *P* < 0.05

**Table 7:** Comparison of SRS-22 outcome between two groups

		**Adding-on group(*n* = 8)**	**No adding-on group(*n* = 38)**	*P* value
**Function, activity**	Preoperative	4.3 ± 0.3	4.3 ± 0.3	0.809
	Postoperative 24-month follow-up	4.4 ± 0.3	4.4 ± 0.3	0.853
**Pain**	Preoperative	4.0 ± 0.4	4.0 ± 0.3	0.618
	Postoperative 24-month follow-up	4.2 ± 0.2	4.1 ± 0.3	0.540
**Self-image, Appearance**	Preoperative	3.6 ± 0.2	3.6 ± 0.4	0.324
	Postoperative 24-month follow-up	4.1 ± 0.2	4.2 ± 0.2	0.353
**Mental Health**	Preoperative	4.2 ± 0.2	4.1 ± 0.2	0.181
	Postoperative 24-month follow-up	4.2 ± 0.2	4.3 ± 0.2	0.876
**Satisfaction**	Preoperative	3.8 ± 0.4	3.8 ± 0.5	0.989
	Postoperative 24-month follow-up	4.4 ± 0.2	4.2 ± 0.2	0.079
**Total**	Preoperative	4.0 ± 0.1	4.0 ± 0.1	0.876
	Postoperative 24-month follow-up	4.3 ± 0.1	4.2 ± 0.1	0.722

*SRS* Scoliosis Research Society

### Complications

**Fig. 1:**
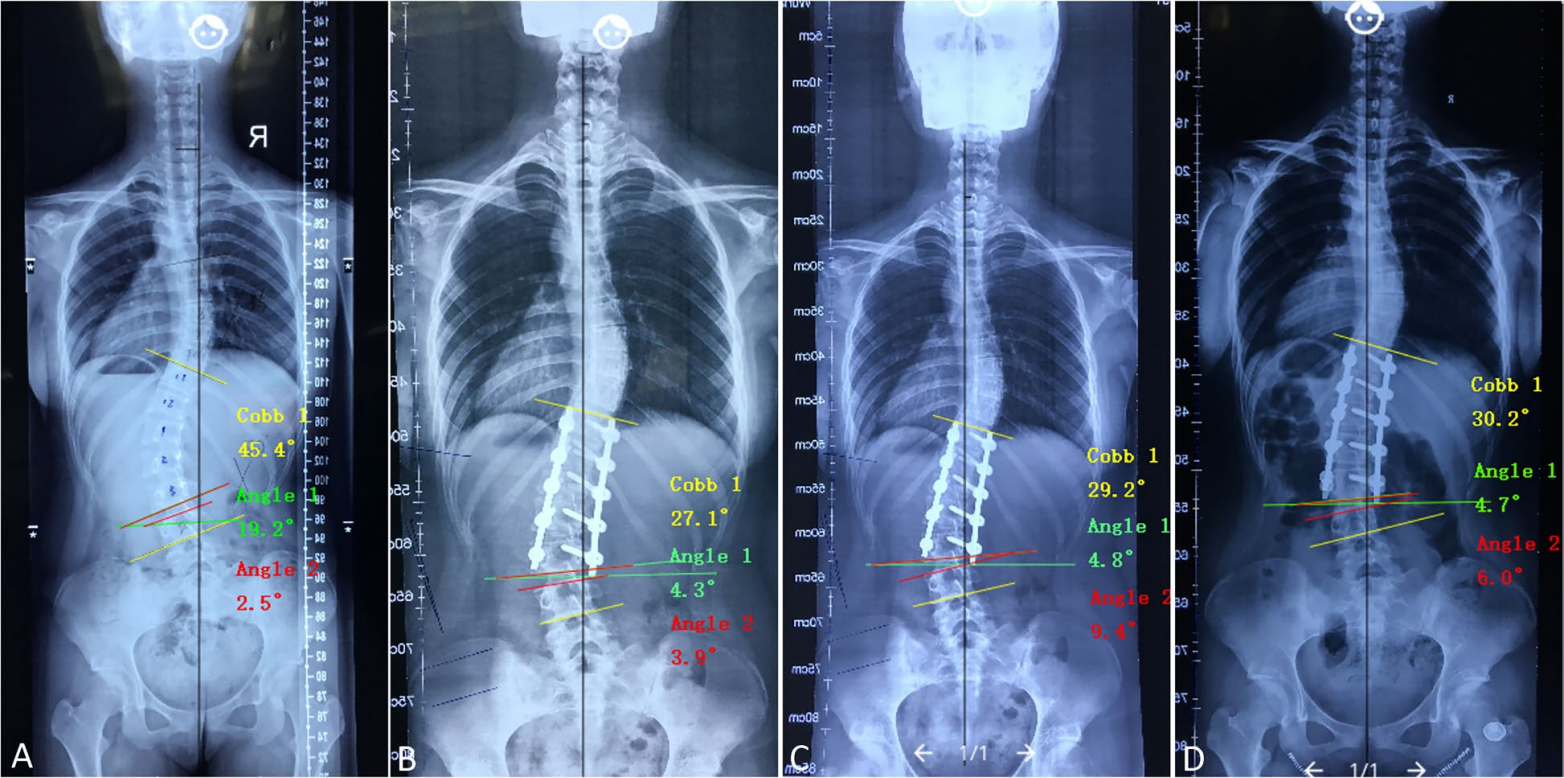
**A** Preoperative standing anteroposterior (AP) radiograph of a 14-year-old female patient with Lenke 5C AIS showed a structural thoracolumbar curve from T11 to L4. The preoperative Cobb angle was 45.4°, L3 tilt was 19.2°, and L3 disc angle was 2.5°. **B** One-week postoperative standing AP radiograph showed the patient underwent posterior fusion from T11 to L3. The Cobb angle was 27.1°, lower instrumented vertebra (LIV) tilt was 4.3°, and LIV disc angle was 3.9°. **C** 3-month postoperative standing AP radiograph. The Cobb angle was 29.2°, LIV tilt was 4.8°, and LIV disc angle was 9.4°. **D** 24-month postoperative standing AP radiograph with brace treatment for 12 months since 3-month after the surgery. The Cobb angle was 30.2°, LIV tilt was 4.7°, and LIV disc angle was 6.0°

There were no intraoperative neuromonitoring alerts, neurologic complications, or incision infections in any of the patients in the present study. Two patients developed mild pulmonary infections, one patient was readmitted due to delayed wound healing, and eight patients had distal adding-on during follow-up. A typical case with distal adding-on was listed (Fig. 1). None of the patients required revision surgery.

## Discussion

In previous studies, it has been shown that inappropriate LIV selection, larger LIV translation, and skeletal immaturity were common risk factors for postoperative distal adding-on in patients diagnosed as Lenke 1A and 2A AIS [15, 20, 21]. Therefore, during the surgical procedures, it may be critical to horizontalize the LIV and minimize the LIV translation to prevent postoperative distal adding-on [21].

The relationship between radiographic parameters and postoperative distal adding-on in patients with Lenke 5C AIS remains uncertain. It is of great importance to determine the LIV to minimize postoperative coronal decompensation. Although L3 or L4 should not be considered as the criteria for LIV selection, most patients with Lenke 5C AIS undergo correction surgery fusing to L3 or L4 [12, 13, 22]. Chang et al[22]. reported that the LIV should be located at L3 in patients with Lenke 5C AIS if L3 is touched by the CSVL, or else the LIV should be located at L4. Kim et al[23]. also reported that the LIV should be fused to L3 when L3 crossing the CSVL. When L4 was selected as the LIV, more fused segments were included, while no better LIV tilt correction or global coronal alignment could be achieved [4].

Shu et al[13]. reported that it is of great importance to horizontalize the LIV and minimize the LIV translation to prevent postoperative distal adding-on in patients with Lenke 5C AIS. Postoperative LIV tilt, LIV disc angle, and LIV translation were confirmed to be risk factors of postoperative distal adding-on, and horizontalization of the LIV would decrease the occurrence of postoperative distal adding-on [13]. Furthermore, it was revealed that the both postoperative LIV tilt and postoperative LIV translation were risk factor of distal adding-on according to the logistic regression analysis [13]. Phillips et al[7]. found that if L3 translation is below 35 mm, L3 may be an ideal LIV in patients with Lenke 5C AIS. Wang et al[12]. suggested that a preoperative LIV translation less than 28 mm and an LIV tilt less than 25° could be confirmed as the criteria for LIV selection. Li et al[24]. found that patients with a preoperative LIV tilt greater than 25° and postoperative LIV tilt more than 8° were of higher risk of coronal imbalance. When the presumed LIV tilt is more than 25°, it has been recommended to fuse to one more level distal [24].

In the present case series, preoperative LIV tilt, LIV disc angle, LIV tilt, LIV translation, and LIV + 1 translation were not significantly different between the two groups. However, preoperative LIV tilt was higher in the adding-on group than in the no adding-on group. In addition, the preoperative LIV translation and LIV + 1 translation of the adding-on group were significantly greater than 25 mm. Perhaps studies including higher numbers of cases could further validate the above conclusions. Therefore, to prevent the occurrence of postoperative distal adding-on, preoperative LIV translation should be less than 25 mm, and postoperative LIV tilt should be less than 8° [17, 24]. LIV rotation has also been thought to be a risk factor in Lenke 5C AIS, and it was advised that the Nash-Moe rotation should be equal to or less than grade I on standing AP radiographs [8, 13].

Barsi et al[10]. reported that intraoperative LIV tilt and LIV disc angle could be measured using prone fluoroscopy, and postoperative LIV tilt and LIV disc angle could be measured using full length AP radiographs during follow-up.

Preoperative LIV translation is of great importance to determine and horizontalize the LIV, thereby optimizing postoperative coronal balance. When the vertebral column is derotated and translated three-dimensionally during the surgical procedure, the touched vertebra can potentially be horizontalized and pulled to the center. Moreover, the criterion of “the LIV should be touched by the CSVL” can be technically more reliable [19]. Based on the present study, the LBTV or LSTV should be selected as the LIV. In cases where the LEV is not touched by the CSVL, LEV + 1 should be evaluated to determine whether it is appropriate to be selected as the LIV.

There are some limitations to the present study. First, the results are limited by the study’s retrospective design. Data were collected prospectively with few cases in the adding-on group, increasing statistical bias. Second, this was a single-center case series, and further validation of multi-center studies may be necessary. Third, the follow-up period was relatively short, it was reported that there may be aggravated coronal imbalance and differing SRS-22 questionnaire outcomes during a 5-year postoperative follow-up.

In conclusion, when the LIV was selected as LBTV-1 or LSTV-1, more postoperative distal adding-on phenomenon may be observed in patients with Lenke 5C AIS. Age at surgery, along with postoperative TL/L curve, LIV translation, LIV + 1 translation, and coronal imbalance, were risk factors for postoperative distal adding-on in these patients.

## Data Availability

The data sets supporting the conclusion of this article are included in the manuscript. Upon request, raw data can be provided by the corresponding author.

## Abbreviations

AIS: Adolescent idiopathic scoliosis
TL: Thoracolumbar
L: Lumbar
LIV: Lower instrumented vertebra
LEV: Lowest end vertebra
LBTV: Last barely touched vertebra
LSTV: Last substantial touched vertebra
CSVL: Central sacrum vertical line
SRS: Scoliosis Research Society
